# N-Methyl-D-Aspartate (NMDA) Receptor Encephalitis Without Electroencephalogram Abnormalities or Cerebrospinal Fluid Pleocytosis Associated With a Tiny Ovarian Teratoma: A Case Report

**DOI:** 10.7759/cureus.93699

**Published:** 2025-10-02

**Authors:** Akihiro Miyashita, Kensuke Takaoka, Jatinder Lachar

**Affiliations:** 1 Department of Internal Medicine, John A. Burns School of Medicine, University of Hawai'i, Honolulu, USA; 2 Department of Internal Medicine, University of Iowa Health Care, Iowa City, USA; 3 Department of Internal Medicine, University of California, Los Angeles, USA

**Keywords:** autoimmune disorder, electroencephalogram, mature cystic ovarian teratoma, nmda-receptor encephalitis, ovarian teratoma

## Abstract

N-methyl-D-aspartate (NMDA) receptor encephalitis is one of the autoimmune disorders characterized by neuropsychiatric symptoms, most commonly associated with ovarian teratoma. While detection of NMDA receptor antibody in cerebrospinal fluid (CSF) is the diagnostic standard, it is often limited by a delayed turnaround time. Abnormal electroencephalogram (EEG) findings and pleocytosis in CSF analysis are frequently observed, and computed tomography (CT) can aid in identifying an underlying ovarian teratoma. Here we report the case of a 36-year-old female who presented with acute psychosis, had a normal EEG and no CSF pleocytosis, and was later diagnosed with NMDA receptor encephalitis associated with a 7 mm ovarian teratoma. This case underscores the diagnostic challenges in the setting of a normal EEG and no CSF pleocytosis, which emphasizes the importance of NMDA receptor antibody testing in CSF for accurate diagnosis.

## Introduction

N-methyl-D-aspartate (NMDA) receptor encephalitis is a rare autoimmune disorder caused by IgG1 antibodies leading to internalization and functional disruption of synaptic NMDA receptors. This results in decreased receptor density and subsequent neuropsychiatric symptoms [1,2]. It predominantly affects children and young female adults, and most cases are associated with ovarian teratoma [3]. Clinical presentation is characterized by a prodrome of fever, headache, or malaise followed by psychiatric symptoms, memory deficits, seizures, and decreased consciousness [4,5]. While the gold standard for diagnosis is the detection of NMDA-receptor antibody in cerebrospinal fluid (CSF), the results often take time to return [3]. Typical electroencephalogram (EEG) features seen in patients with NMDA receptor encephalitis are diffuse or focal slowing of background activity, and a normal EEG finding is rarely observed [6,7]. Furthermore, CSF analysis typically shows mild to moderate lymphocytic pleocytosis. In this report, we present a rare case of NMDA receptor encephalitis associated with a tiny ovarian teratoma, notably characterized by normal EEG findings and no CSF pleocytosis.

## Case presentation

**Figure 1:**
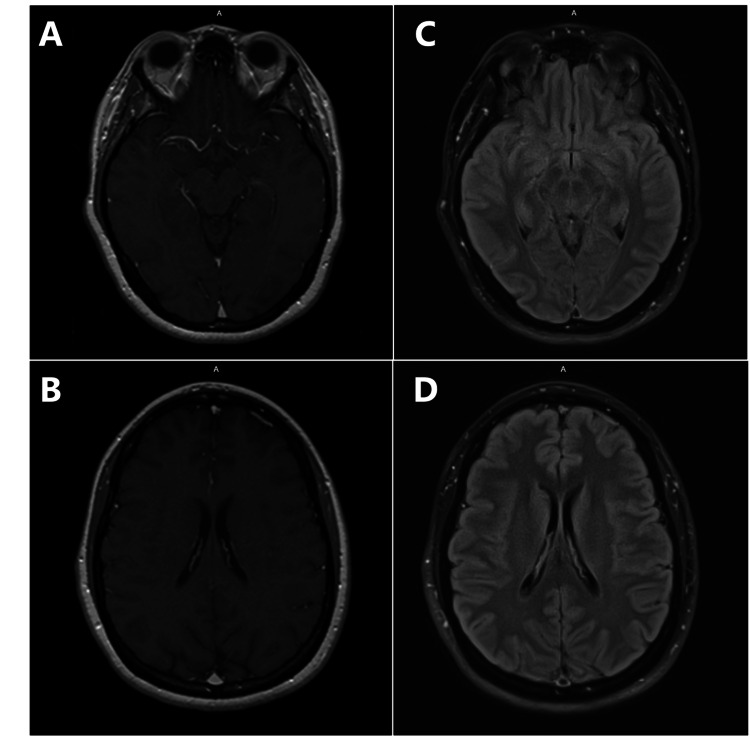
Radiological findings of the brain. Magnetic resonance imaging on T1 (A and B) and fluid-attenuated inversion recovery (FLAIR) (C and D) showed no acute infarct, hemorrhage, or mass lesion. The ventricles, cisterns, and sulci were not effaced.

A 36-year-old female with a history of left ovarian dermoid status post left oophorectomy presented with an acute onset of psychosis. Her symptoms were characterized by fluctuating psychosis with agitation and memory deficits. The laboratory results, including complete blood count, electrolytes, renal and liver function tests, vitamin B12, folate, ammonia, HIV, syphilis, and thyroid function, were unremarkable. Urine drug screens were negative except for the positive result for tetrahydrocannabinol. The patient was initially admitted to the psychiatry unit and treated with valproate, lorazepam, and ziprasidone for a presumed primary psychiatric disorder, but there was no significant improvement. Therefore, the patient was transferred to the internal medicine unit for neurological workup. EEG showed a normal background associated with one episode of psychogenic non-epileptic seizure. Computed tomography (CT) of the head and magnetic resonance imaging (MRI) of the brain showed no acute abnormalities (Figure 1). 

CSF analysis demonstrated no white blood cells, glucose of 53 mg/dL, protein of 51 mg/dL (normal range: 15-45 mg/dL), and negative results on the meningitis PCR panel for *E. coli* K1, *Haemophilus influenzae*, *Listeria*, *Neisseria*, *Streptococcus agalactiae*, *Streptococcus pneumoniae*, cytomegalovirus, enterovirus, herpes simplex virus types 1 and 2, human herpesvirus 6, varicella, human parechovirus, and *Cryptococcus*. Serum autoimmune screening showed positive NMDA-receptor antibody (Table 1), whereas serum NMDA-receptor antibody titer was negative (less than 1:240). Vaginal ultrasound showed a 10 mm echogenic nodule in the right ovary. CT of the chest, abdomen, and pelvis revealed no evidence of malignancy, including an ovarian mass. The patient was empirically treated with a five-day course of intravenous immunoglobulin (0.4 mg/kg/day) and electroconvulsive therapy, leading to partial improvement of her symptoms. Subsequently, the CSF paraneoplastic panel came back positive for NMDA-receptor antibody (Table 2).

**Table 1:** Serum autoimmune panel only detected NMDA-R antibody Ab, antibody; IFA, immunofluorescence assay; CBA, cell-based assay; NMDA-R, n-methyl-d-aspartate receptor; AMPA-R, α-amino-3-hydroxy-5-methyl-4-isoxazolepropionic acid receptor; GAD, glutamic acid decarboxylase; GFAP, glial fibrillary acidic protein; CASPR2, contactin-associated protein-like 2; LGI1, leucine-rich glioma inactivated 1; CRMP-5, collapsin response mediator protein 5; DPPX, dipeptidyl-peptidase-like protein 6; IGLON5, immunoglobulin-like cell adhesion molecule 5; PDE10A, phosphodiesterase 10A; TRIM46, tripartite motif-containing 46

Serum laboratory analysis	Result
AMPA-R	Negative
Amphiphysin	Negative
Anti-Glial Nuclear Ab, Type 1	Negative
Anti-Neuronal Nuclear Ab, Type 1	Negative
Anti-Neuronal Nuclear Ab, Type 2	Negative
Anti-Neuronal Nuclear Ab, Type 3	Negative
CASPR2-IgG	Negative
GABA-B-R Ab	Negative
GAD65 Ab	Negative
GFAP IFA	Negative
LGI1-IgG	Negative
mGluR1 Ab	Negative
NIF IFA	Negative
NMDA-R Ab	Positive
Purkinje Cell Cytoplas. Ab, Type 1	Negative
Purkinje Cell Cytoplas. Ab, Type 2	Negative
Purkinje Cell Cytoplas. Ab, Type Tr	Negative
CRMP-5-IgG	Negative
Neurochondrin IFA	Negative
Septin-7 IFA	Negative
DPPX Ab CBA	Negative
IGLON5 CBA	Negative
PDE10A Ab IFA	Negative
TRIM46 Ab IFA	Negative

**Table 2:** Cerebrospinal fluid (CSF) encephalitis panel detecting NMDA-R antibody. Ab, antibody; IFA, immunofluorescence assay; CBA, cell-based assay; NMDA-R, n-methyl-d-aspartate receptor; AMPA-R, α-amino-3-hydroxy-5-methyl-4-isoxazolepropionic acid receptor; AGNA-1, anti-glial nuclear antibody type 1; ANNA, anti-neuronal nuclear antibody; GAD, glutamic acid decarboxylase; GFAP, glial fibrillary acidic protein; CASPR2, contactin-associated protein-like 2; LGI1, leucine-rich glioma inactivated 1; CRMP-5, collapsin response mediator protein 5; DPPX, dipeptidyl-peptidase-like protein 6; IGLON5, immunoglobulin-like cell adhesion molecule 5; PDE10A, phosphodiesterase 10A; TRIM46, tripartite motif-containing 46

CSF laboratory analysis	Result
AMPA-R Ab	Negative
Amphiphysin	Negative
AGNA-1	Negative
ANNA-1	Negative
ANNA-2	Negative
ANNA-3	Negative
CASPR2-IgG	Negative
CRMP-5	Negative
GABA-B-R Ab	Negative
GAD65 Ab	Negative
GFAP IFA	Negative
LGI1-IgG	Negative
mGluR1 AB IFA	Negative
NIF IFA	Negative
NMDA-R Ab	Positive
Purkinje Type Tr	Negative
PCA-1	Negative
PCA-2	Negative
Neurochondrin IFA	Negative
Septin-7 IFA	Negative
DPPX Ab CBA	Negative
IGLON5 CBA	Negative
PDE10A Ab IFA	Negative
TRIM46 Ab IFA	Negative

**Figure 2:**
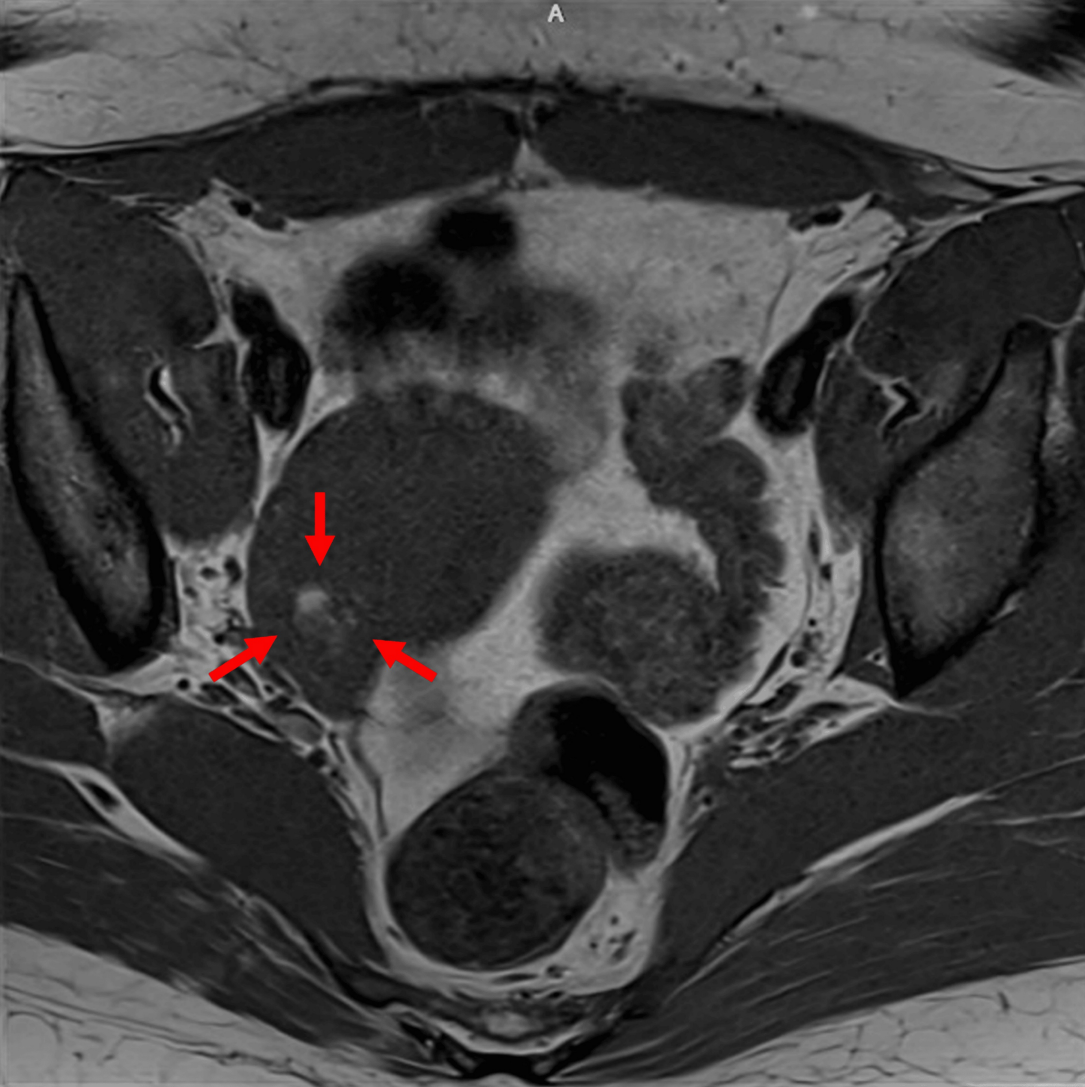
Radiological findings of ovarian teratoma. Magnetic resonance imaging showed a small fatty lesion within the central right ovary without enhancement (red arrow).

**Figure 3:**
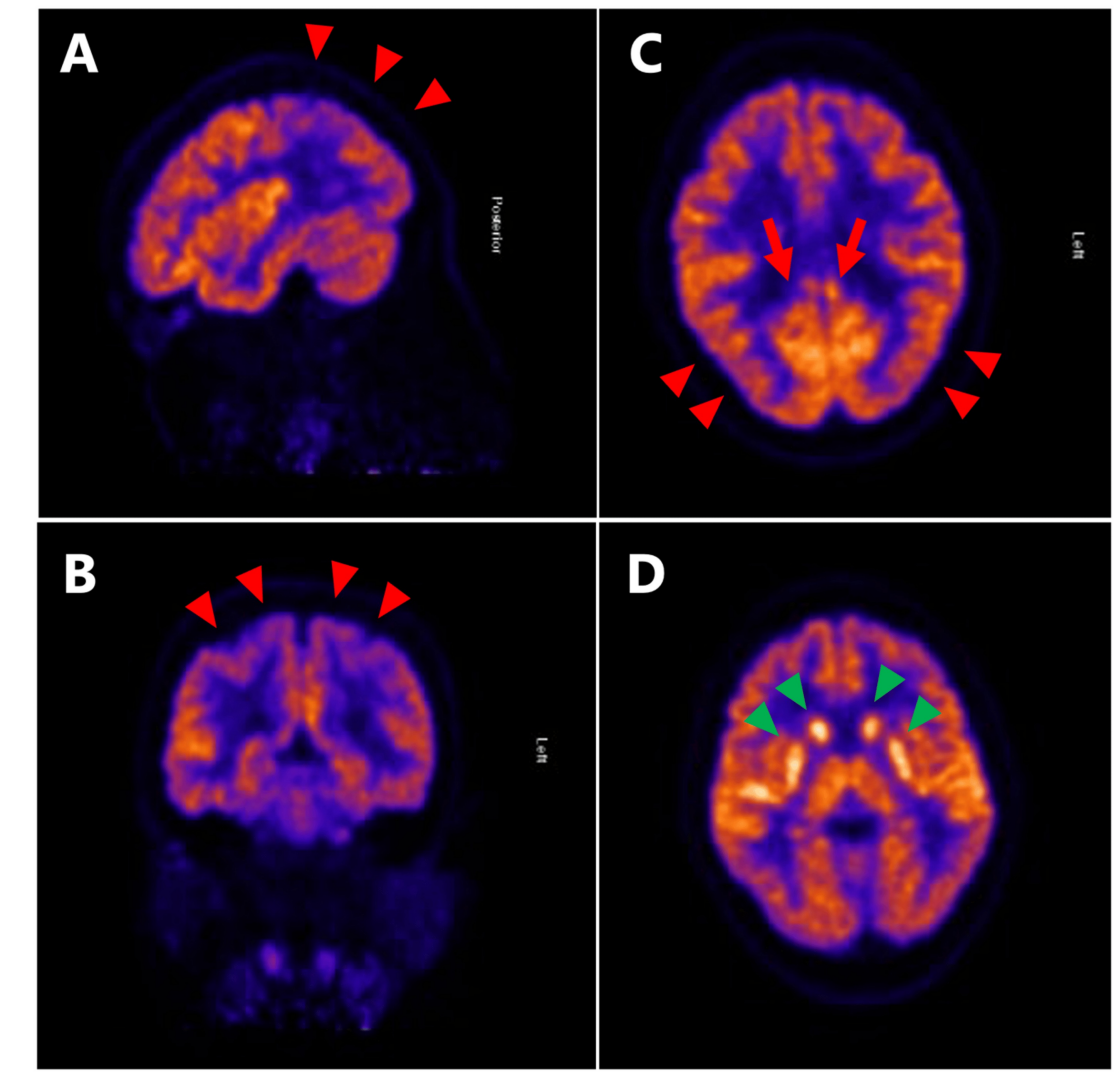
Fluorodeoxyglucose positron emission tomography (FDG-PET) findings of the brain. There is significantly reduced glucose metabolism in the parietal lobes (A, B, and C, red arrowheads), extending into the occipital lobes while sparing the visual cortex (C, red arrow). There is increased metabolism in the caudate, putamen, and thalamus (D, green arrowheads).

An MRI pelvis scan detected a possible 7 mm fatty lesion within the central right ovary without enhancement, representing a small dermoid lesion (Figure 2). Fluorodeoxyglucose positron emission tomography (FDG-PET) did not show evidence of malignancy. Additionally, FDG-PET of the brain (Figure 3) demonstrated significantly reduced glucose metabolism in the parietal lobes with extension into the occipital lobes, sparing the visual cortex, and in the lateral frontal lobes bilaterally. There was also hypermetabolic activity in the caudate, putamen, thalamus, and superior temporal cortices. These observations aligned with NMDA-receptor encephalitis. The patient had a right salpingo-oophorectomy, followed by rituximab therapy. The pathology result was consistent with an ovary with a mature cystic teratoma. Patient’s symptoms gradually improved after these treatments.

## Discussion

We reviewed a case of NMDA receptor encephalitis without EEG abnormalities or CSF pleocytosis, associated with a 7 mm ovarian teratoma. NMDA receptor encephalitis is a rare autoimmune encephalitis, commonly associated with ovarian teratoma [1]. Fundamental treatment includes surgical removal of the detected tumor and immunosuppressive therapy, including high-dose corticosteroids, intravenous immunoglobulin (IVIG), or plasma exchange [3]. In our case, surgical removal and immunosuppressive therapy, including IVIG and rituximab, improved the symptoms.

Diagnosis of NMDA receptor encephalitis can sometimes be challenging. In this case, the patient’s psychosis was initially attributed to a primary psychiatric disorder. However, because her agitation and memory deficits failed to improve with psychiatric management, we proceeded with an expanded diagnostic workup. EEG is abnormal in most cases, typically showing non-specific, slow, and disorganized activity with electrographic seizures [3], and a normal EEG is reported in only about 4% of cases [7]. This case showed a normal EEG with one episode of psychogenic non-epileptic seizure, which made the diagnosis difficult. The reason for the normal EEG is unclear, but one possible explanation is that it was obtained during the early phase of the disease. One retrospective analysis reported that 20% of patients had normal EEGs within the first 14 days of symptom onset [6]. However, this analysis included only 10 patients at the early stage, underscoring the need for further investigation into EEG findings during the initial phase of the disease.

While detection of NMDA-receptor antibody in CSF is the gold standard for diagnosis and has a higher sensitivity (99%) compared to serum (68%), it often requires a prolonged turnaround time [8]. Although some studies suggested that antibody titers may correlate with disease severity, the presence of antibody is a key diagnostic clue even at low titers [9]. In our case, the NMDA-receptor antibody was detected in the serum, but the titer was negative, initially raising concern for a false-positive result. Furthermore, CSF analysis typically shows mild to moderate lymphocytic pleocytosis ranging from 10 to 100 cells/microL, especially in the early stages of the disease [10-13]. Additionally, mild elevation of CSF protein is also observed in 60% of patients [12]. These CSF abnormalities occur in 91%-94% of cases, while they might be normal early in the disease course [2,13]. In the present case, low titers of serum NMDA-receptor antibody and the absence of pleocytosis in CSF further complicated the diagnostic process.

In NMDA receptor encephalitis, brain FDG-PET typically shows relative hypermetabolism in the frontal and temporal cortices, marked hypometabolism in the parietal and occipital lobes, and hypermetabolism in the basal ganglia, with these patterns correlating with disease severity [14,15]. In our case, hypermetabolism of the frontal lobes was not seen, but hypometabolism in the parietal and occipital lobes, with increased metabolism of the basal ganglia, was consistent with NMDA receptor encephalitis. The basal ganglia play a crucial role in emotional regulation, and dysfunction in this region may have contributed to the development of paranoia in this case. In general, FDG-PET can provide results relatively quickly and may serve as a useful diagnostic tool to guide empiric treatment, particularly when symptoms are too severe to be managed with supportive care alone. Therefore, these FDG-PET findings may also become an important diagnostic tool, especially when other modalities such as CSF or EEG are inconclusive.

## Conclusions

In conclusion, we present a rare case of NMDA receptor encephalitis with normal EEG and no pleocytosis, possibly caused by a tiny ovarian teratoma. Further research is warranted to clarify the relationship between ovarian teratoma and encephalitis.

## References

[1] Dalmau J, Graus F (2018). Antibody-mediated encephalitis. N Engl J Med.

[2] Dalmau J, Gleichman AJ, Hughes EG (2008). Anti-NMDA-receptor encephalitis: case series and analysis of the effects of antibodies. Lancet Neurol.

[3] Dalmau J, Lancaster E, Martinez-Hernandez E, Rosenfeld MR, Balice-Gordon R (2011). Clinical experience and laboratory investigations in patients with anti-NMDAR encephalitis. Lancet Neurol.

[4] Xu J, Zhao N, Guan H, Walline JH, Zhu H, Yu X (2022). Anti-N-methyl-D-aspartate receptor encephalitis: characteristics and rapid diagnostic approach in the emergency department. BMC Neurol.

[5] Gurrera RJ (2019). Frequency and temporal sequence of clinical features in adults with anti-NMDA receptor encephalitis presenting with psychiatric symptoms. Psychol Med.

[6] Zhang Y, Liu G, Jiang MD, Li LP, Su YY (2017). Analysis of electroencephalogram characteristics of anti-NMDA receptor encephalitis patients in China. Clin Neurophysiol.

[7] Sonderen AV, Arends S, Tavy DL (2018). Predictive value of electroencephalography in anti-NMDA receptor encephalitis. J Neurol Neurosurg Psychiatry.

[8] Bastiaansen AE, de Bruijn MA, Schuller SL (2022). Anti-NMDA receptor encephalitis in the Netherlands, focusing on late-onset patients and antibody test accuracy. Neurol Neuroimmunol Neuroinflamm.

[9] Zandi MS, Paterson RW, Ellul MA (2015). Clinical relevance of serum antibodies to extracellular N-methyl-D-aspartate receptor epitopes. J Neurol Neurosurg Psychiatry.

[10] Wu S, Li H, Lian Y (2020). Anti-N-methyl-D-aspartate receptor encephalitis: a prospective study focused on cerebrospinal fluid and clinical symptoms. Neurol Sci.

[11] Liba Z, Kayserova J, Elisak M (2016). Anti-N-methyl-D-aspartate receptor encephalitis: the clinical course in light of the chemokine and cytokine levels in cerebrospinal fluid. J Neuroinflammation.

[12] Zrzavy T, Höftberger R, Wimmer I, Berger T, Rommer P, Macher S (2021). Longitudinal CSF Findings in Autoimmune Encephalitis-A Monocentric Cohort Study. Front Immunol.

[13] Dürr M, Nissen G, Sühs KW (2021). Cerebrospinal fluid findings in acute NMDA receptor and LGI1 antibody-associated autoimmune encephalitis. Neurol Neuroimmunol Neuroinflamm.

[14] Kalra S, Tripathi M, Tripathi M (2024). Role of FDG PET/CT in definitive and presumed autoimmune encephalitis. Nucl Med Commun.

[15] Leypoldt F, Buchert R, Kleiter I (2012). Fluorodeoxyglucose positron emission tomography in anti-N-methyl-D-aspartate receptor encephalitis: distinct pattern of disease. J Neurol Neurosurg Psychiatry.

